# Local Renin-Angiotensin System in the Reproductive System

**DOI:** 10.3389/fendo.2013.00150

**Published:** 2013-10-18

**Authors:** Daniel Herr, Inga Bekes, Christine Wulff

**Affiliations:** ^1^Department of Obstetrics and Gynaecology, University of Saarland, Homburg, Germany; ^2^Department of Obstetrics and Gynaecology, Ulm University Medical Centre, Ulm, Germany

**Keywords:** endometrial cancer, endometrium, ovarian cancer, ovary, renin-angiotensin system, reproductive tract, local

## Abstract

The renin-angiotensin system (RAS) is well known as regulator of electrolytes and blood pressure. Besides this function, there are numerous studies supporting the idea of a local tissue RAS. This system controls the local activity of the different RAS family members, especially of the functional proteins Angiotensin II and Angiotensin (1–7). Those antagonistically acting proteins have been described to be expressed in different organ systems including the human reproductive tract. Therefore, this local RAS has been suspected to be involved in the control and regulation of physiological and pathological conditions in the female reproduction tract. This review of the available literature summarizes the physiological influence of the RAS on the follicular development, ovarian angiogenesis, and placental- and uterine function. In addition, in the second part the role of the RAS concerning ovarian- and endometrial cancer becomes elucidated. This section includes possible novel therapeutic strategies via inhibition of RAS-mediated tumor growth and angiogenesis. Looking at a very complex system of agonistic and antagonistic tissue factors, it may be supposed that the RAS in the female reproduction tract will be of rising scientific interest in the upcoming years.

## Introduction

The Renin-Angiotensin system (RAS) is of paramount importance for the perpetuation of the circular flow, regulating the electrolyte metabolism thus the blood pressure (1–4). This considerable function of the RAS is mediated by the systemic RAS-pathway. The latter consists of a cascade of peptides, acting as precursors which become transformed by different enzymes into the bioactive end products (5). The main protein of this system is Angiotensinogen, which is synthesized in the liver (6). Following the pathway, Angiotensinogen becomes converted into Angiotensin I, catalyzed by Renin, which is of renal origin. Subsequently, Angiotensin I can be further modulated by the angiotensin-converting enzyme (ACE) I to Angiotensin II or by ACE II to Angiotensin (1–9).

Angiotensin (1–9) then becomes transformed by ACE or neutral endopeptidase (NEP) into Angiotensin (1–7) (7–9). Those two bioactive effector molecules, Angiotensin II and Angiotensin (1–7) act in an antagonistic way by binding to different receptors: angiotensin receptor type 1 (AT1R) and type 2 (AT2R) or Mas-receptor (10). The G-coupled Mas-receptor mediates vasodilatory and anti-proliferative effects and antagonizes actions of the AT1R (11). In recent years, attention has also focused on the evidence of a widespread local tissue RAS (12). Expression of elements of this local RAS has been described in different parts of the human reproductive tract. Apparently, the both antagonistic bioactive proteins of the RAS, in particular Angiotensin II and Angiotensin (1–7) can result from the local tissue RAS (13, 14). This local production of the bioactive peptides is not necessarily dependent on the local expression of all components of the local tissue RAS, since it is also possible to take up components from the circulation, such as renin. Furthermore, besides presence of Angiotensin II and Angiotensin (1–7), expression of AT1R, AT2R, and Mas-receptor human reproductive tissue is needed in order to mediate the local impact of the RAS for physiological and pathological processes, including follicle maturation, fine-tuning of the regulation of reproduction, angiogenesis as well as tumor cell proliferation (15–18). An influencing effect on cancer has been described for different tumor types already during the last two decades (19, 20).

## Materials and Methods

We performed a systematic literature review concerning presence and function of the RAS in the female reproduction tract. This was based on the medical databases Medline, Embase, BIOSIS, and CINHAL. Literature analysis was conducted without a timeframe on all existing publications including 2013. All manuscripts were sighted based on the title and abstract and any duplicate manuscripts occurring in the literature search were excluded. After fulfilling the inclusion criteria (content-related, experimental, and clinical studies, in the case of experimental studies dividing into studies in humans and/or animals) the manuscripts were reviewed and analyzed. Thereby, the data was extracted and content-related articles allocated into two different groups:

- Physiological role of the RAS in the reproductive tract?
- Role of the RAS in gynecologic cancers.

## Physiological Role of the RAS in the Reproductive Tract

### Ovary and follicular development

Definitely, a local tissue RAS is present in the adult ovary (18). In the human ovary, all family members of the RAS have been proven at the protein level, whereas in the bovine, porcine, and rat ovary only single compounds of the RAS are expressed (12, 21–23). It has been speculated, that the attendance of RAS compounds is significantly involved in the regulation of fetal development since RAS expression can be observed in the porcine ovary already around 45 days of gestation: AT1R and AT2R have been detected in granulosa cells of primordial, primary, and secondary follicles (23). In addition, Angiotensin II and its receptors AT1R/AT2R seem to have regulatory effects in the ovary regarding oocyte nuclear maturation and ovulation (24–27). This regulative function has mainly been investigated in antral follicles, but also in porcine granulosa cells of earlier stages of follicular development (28). Obviously, there are significant differences between the species. When bovine Cumulus oocyte complexes (COCs) were cultivated with Angiotensin II, nuclear maturation of the oocyte was induced (12, 26). Furthermore, Ferreira et al. indicated that Ang II may have an impact on bovine ovulation via AT2R (25). In addition, functional studies have demonstrated that inhibition of the AT2R prevents bovine ovulation significantly (29). In rats, AT1R is expressed in healthy follicles (30) and AT2R-expression is obviously involved in follicular atresia through apoptosis (31, 32).

Unfortunately, the published data concerning involvement of the RAS in the regulation of the hormonal regulation of the ovary is scanty and sometimes inconsistent. For example, for the bovine corpus luteum, it has been shown, that tissue levels of Angiotensin II do not change throughout the cycle, indicating that steroids may have no influence on tissue RAS (33). In conflict with this finding, a significant influence of the RAS on progesterone synthesis has been described (34, 35). The observed increase of progesterone and soluble and membrane-bound aminopeptidase A was accompanied by a decrease of membrane-bound aminopeptidases B/N (RAS-regulating enzymes) due to inhibition alpha 1-adrenergic receptors in rats (35). In addition, the data concerning gonadotropin-dependent expression of RAS-proteins is disputed: it has been shown that application of hCG in case of early pregnancy has the capacity to activate the local RAS in the ovary (36), whereas our own group observed a significant hCG-dependent decrease of Angiotensin II in human granulosa lutein cells *in vitro* (37). This result goes in line with the perception that the antagonistically to Angiotensin II acting Angiotensin (1–7) and its receptor Mas were found to be increased after gonadotropin stimulation in the rat ovary (22). Basically, the role of Angiotensin (1–7) seems to be of increasing interest: Angiotensin (1–7), Mas-receptor, and ACE 2 were identified in all stages of follicular development in humans (38) and functional studies indicate a role of the Angiotensin (1–7)-pathway in the rodent *in vivo* (39) suggesting to be a mediator of gonadotropin functions in the ovulatory cascade (40).

### Ovarian vasculature function and angiogenesis

The most outstanding data in the literature has been published concerning the regulatory character of the RAS on vascular function and angiogenesis in the ovary. The invoking effects on the vessels are thereby first of all adapted from the Angiotensin II-AT1R-pathway (41–44). To be contrary to this, the restitution of the luteal vasculature is mediated by the AT2R-pathway (45). Anyway, Angiotensin II obviously influences the microvascular endothelial function in the corpus luteum (42). Hayashi et al. demonstrated that microvascular endothelial cells (MVE) in the corpus luteum express ACE and are capable to convert Angiotensin I into Angiotensin II. The Angiotensin II production thereby increases significantly under stimulation with estradiol in combination with vascular endothelial growth factor (VEGF) (41). MVE furthermore possess AT1R and AT2R (41, 42). Interestingly, the expression of those two receptors differ throughout the cycle: AT1R remains constant but AT2R-expression is lowest during the mid luteal phase and highest during the late luteal phase (41, 46). The regulation of angiogenic processes is urgently needed to ensure the constant flow of growth, maturation, and demise of the corpus luteum. It has been shown by our group, human granulosa lutein cells collected during *in vitro* fertilization (IVF) are expressing several components of the RAS (47). In addition, we demonstrated that exogenous Angiotensin II stimulation increased VEGF synthesis via AT1R signaling *in vitro* (47). This data may implicate the regulatory effect of the RAS on angiogenesis in the corpus luteum. In agreement with its meaning concerning control of systemic blood pressure, the individual family members of the local RAS also regulate perfusion and vascular tone in the ovary (36, 44).

### Placenta

The human placenta is one of the most interesting tissues in the reproductive tract, because the utero-placental unit provides a transposition of nutritive substances and oxygen between mother and fetus. It has been assumed by many authors that the RAS influences the placental function (48–52), since all different components of the RAS are expressed in human placenta cell lines (53) as well in placental tissue (54, 55). However, functional data of the placental RAS is very rare. Obviously, the different RAS-proteins are expressed differentially in the various areas of this organ: angiotensinogen, Renin, Angiotensin I, Angiotensin II, ACE, AT1R, and AT2R were localized to maternal decidua (56, 57) and Angiotensin II and ACE were additionally found in pericytes of endometrial spiral arteries. However, Angiotensinogen and renin also have been detected in fetal capillaries (58). The AT1R, which is predominantly expressed in the placenta, was found in cytotrophoblast and syncytiotrophoblast cells as well as in fetal capillaries, while little is known concerning localization of the AT2R (59, 60). The antagonistic proteins to Angiotensin II, namely Angiotensin (1–7) and ACE2 were found to be expressed in syncytiotrophoblast, cytotrophoblast, and vascular smooth muscle cells of primary and secondary villi (58). The above mentioned members of the RAS family can be detected from 6 weeks of gestation until birth. Obviously, there are some variations in the course of pregnancy: it has been shown that mRNA of ACE is increasing during gestation but decreases near term. In addition, AT1R mRNA and AT1R protein levels are rising throughout the entire pregnancy, reaching highest levels at the end (61). Since a direct connection between Angiotensin II and AT1R has been observed in the placenta, it has been supposed that this fact indicates a regulating effect of Angiotensin II on the AT1R expression (62). From a more clinical point of view, there are several references that the placental RAS is involved in trophoblast invasion and angiogenesis (63, 64), being a possible cause of defect for the development of conditions with disordered utero-placental perfusion, namely preeclampsia (see below).

### Fallopian tube

Data concerning the RAS and the oviduct is rare. Any clinical relevant findings have not been published. However, Angiotensin II has been localized in blood vessel endothelium and in stromal cells. Both binding and Angiotensin II type-2 receptor mRNA were detected at high levels, but no differences in receptor concentration could be detected in fallopian tubes ipsilateral or contralateral to the corpus luteum (65).

### Uterus

Outline above, the data of uterine RAS are of descriptive nature and mostly limited to the endometrium. Studies investigating the functional relevance of the RAS in the uterus are rare.

Being an indispensible part of the reproductive tract, the endometrium underlies a cyclic change of growth and degradation. Basically, all elements of the local tissue RAS are expressed in the endometrium (66), however this expression diversifies during the cycle (46, 67): angiotensin II underlies cyclic variances within the endometrium and is increased during the proliferative phase, and decreased during the secretory phase (68). Angiotensin-(1–7) and its receptor MAS is also present throughout the menstrual cycle but increases in the glandular endometrium in the mid and late secretory phase. Although AT1R and AT2R are expressed in the endometrium, expression of AT2R is more frequent and varying (68) and it is down-regulated during pregnancy (69). The AT2R-expression is thereby most prominent in the myometrium (up to 90%) as compared to AT1R expression (up to 10%). Unfortunately, data concerning functional effects of the RAS in the uterus is rare. Since endometrium is controlled by female sex hormones, it has been supposed that the RAS might also be influenced by those hormones. This assumption is supported by the finding, that the local expression and production of renin is increased after stimulation with progesterone (70).

## Pathophysiological Role of the RAS in the Reproductive Tract

During the past few years, the primary small number of publications concerning pathophysiologic aspects of the RAS has been markedly increased. This affects aspects of reproduction, in essence preeclampsia, as well as of the role of the RAS controlling gynecological cancers. Thereby, the most resilient data is available regarding the regulatory aspects on tumor cell proliferation, vascular function, and angiogenesis (71).

### RAS and reproduction

In patients with polycystic ovary syndrome, the intra-follicular renin, which is needed for synthesis of the bioactive peptides of the RAS, affects maturation and oocyte quality (43). Follicles with high levels of renin indicating a high local RAS activity, were associated with better oocyte quality and showed higher VEGF concentrations during IVF procedures (43). Furthermore, a strong activation of the local ovarian RAS by beta-hCG has been observed during IVF treatment. This process was also associated with an increased VEGF concentration. Consequently, it has been assumed, that the activation of the ovarian RAS and consecutive high levels of VEGF might act synergistically during pathogenesis of ovarian hyperstimulation syndrome (OHSS).

Although the role of the RAS concerning invasion of trophoblast and placentation is poorly investigated, there is evidence that dysfunction of this system may cause hypertension and preeclampsia (56, 72, 73, 74): patients with preeclampsia present with increased expression of Angiotensin II and AT1R in maternal decidua cells and in the placenta itself (74, 75). In pre-eclamptic pregnancies Angiotensin II and AT1R was been observed to be increased, whereas levels of Angiotensin I, Angiotensin (1–7), ACE, and ACE2 were normal as compared to healthy pregnancies (48).

Current data describe a relevant clinical link between RAS and preeclampsia: women with a male fetus who developed gestational hypertension had increased Angiotensin (1–7) levels at 15 weeks gestation compared with women with normal pregnancies, suggesting that these women were on an early trajectory for the development of hypertension. Therefore, the authors proposed measurement of Ang-(1–7) during early pregnancy in order to predict new-onset hypertension (76).

In addition Valdes et al. also reported elevated Angiotensin (1–7) concentrations in spontaneously aborted and ectopic early pregnancy placentas, which lead the authors to hypothesize that the ACE2–Angiotensin-(1–7) axis plays a functional role in placental development (77). Further recent data underlines the connection between RAS and preeclampsia, since there is an association with a polymorphism of Angiotensinogen in Chinese women. This finding might cause disordered vasculogenesis contributing to the development of preeclampsia (78).

## Ovarian Cancer

The published data apropos RAS and invasive epithelial ovarian cancer provides a role concerning proliferation and dissemination of cancer cells and tumor-angiogenesis. Ovarian cancer cells express Angiotensin II and AT1R (79) but there is still missing evidence of the other components of the local RAS in ovarian cancer. It has been shown that levels of AT1R are higher in borderline lesions and in invasive epithelial ovarian cancer as compared with normal ovaries (80). Being in line with this finding, ovarian cancer patients presenting with high levels of AT1R, have a worse prognosis in comparison with tumors lacking the AT1R expression. Obviously, the Angiotensin II → AT1R pathway is able to influence ovarian cancer cell proliferation (80). Since it has been shown that levels of VEGF as well as rates of angiogenesis are increased due to Angiotensin II, the link between the RAS and Angiogenesis has been established in epithelial ovarian cancer cells (80). At least *in vivo*, this connection even acts quantitatively: stimulation of the ovarian cancer cell line Scov 3 with Angiotensin II caused increased VEGF expression (80) and high levels of AT1R are associated with significantly increased VEGF production and micro-vessel density (MVD) (79). All those findings might indicate a therapeutical approach. Therefore, inhibition of AT1R has been performed in mice with peritoneal carcinomatosis, leading to a significant decrease of ascites and peritoneal tumor cell dissemination. In patients, current data at least indicate that agonistic auto antibodies against AT1R may be associated with advanced progression of early ovarian cancer (81). These findings implicate that AT1-AA might be selected as a detectable biomarker and potential therapeutic target in diagnosis and treatment of EOC patients. Summing up, it appears that two substantial mechanisms, increased tumor cell proliferation and angiogenesis are mediated by the RAS. Therefore, targeting the Angiotensin II → AT1R pathway could provide a future treatment strategy for invasive epithelial ovarian cancer.

## Endometrial Cancer

Similarly to ovarian cancer, there are proofs for a possible influence of the local RAS concerning endometrial cancer. It has been published that in endometrial cancer, prognosis, tumor cell proliferation, and angiogenesis are affected by the RAS (82). According to the situation in the ovary, again increased local levels of Angiotensin II are associated with poorer prognosis in endometrial cancer patients (82, 83). This finding might be due to the fact, that those higher levels of Angiotensin II were found in patients with an advanced tumor stage (82). In this study, 81.9% were positive for Ang II and 59.6% positive for the AT1R. However, it seems as not only progression of disease but also an increased risk for developing endometrial cancer might be mediated by the RAS: a polymorphism of ACE has been described to be associated earlier onset of this disease (84). Again, the Angiotensin II → AT1R axis increases VEGF and thereby angiogenesis in a dose-dependent way (83). Recently, it has even speculated that Angiotensin II modulates the VEGF type-2 receptor KDR via AT1R (85). Inversely regarded, the connection of the RAS with angiogenesis is supported by the fact that low Angiotensin II activity is associated with less VEGF and a decreased MVD. This relation was basis for functional experiments: the treatment with the AT1R-blocker Losartan has a anti-proliferative effect in endometrial cancer tissue *in vitro* (86). In summary, high activity of the local RAS in endometrial cancer is associated with higher incidences, earlier onset, and increased rates of angiogenesis. The roles of Ang-(1–7) and the AT2R as well as clinical randomized study data is completely lacking and need to be further investigated.

## Conclusion

This review summarizes the available literature concerning the local tissue RAS in the reproductive tract with regards to physiological and pathological clinical situations. The majority of the published studies remain on a non-functional descriptive level, but nevertheless, a role of the local tissue RAS as regulator in the human reproductive tract van be supposed. Obviously, the RAS affects oocyte maturation and quality, endometrial lining as well as hormone production and may therefore be considered as important system for regulation of physiologic pathways. Furthermore, the published data indicates a potential involvement of the local RAS in affecting physiological angiogenesis in the reproductive tract. Currently, pathologic conditions are better investigated than physiology. The Angiotensin II → AT1R pathway promotes tumor growth and angiogenesis in malignancies, arising new treatment strategies by inhibition of the AT1R. Data concerning stimulation of the antagonistic pathways such as the AT2R or Angiotensin (1–7) pathway as treatment modality for ovarian- or endometrial cancer is lacking. Due to current data, it is clear that most conclusions made are speculative since only a negligible number of functional studies have been conducted and clinical randomized data is missing completely. However, regarding a very complex and variable system of agonistic and antagonistic tissue factors, it may be hypothesized that the RAS in the female reproduction tract will be of increasing interest in the near future.

## Conflict of Interest Statement

The authors declare that the research was conducted in the absence of any commercial or financial relationships that could be construed as a potential conflict of interest.

## References

[1] Carey RM The intrarenal renin-angiotensin and dopaminergic systems: control of renal sodium excretion and blood pressure. Hypertension (2013) 61:673–80 10.1161/HYPERTENSIONAHA.111.00241 23407646 PMC3577093

[2] Herichova I Szantoova K Renin-angiotensin system: upgrade of recent knowledge and perspectives. Endocr Regul (2013) 47:39–52 10.4149/endo_2013_01_39 23363256

[3] Mulrow PJ Angiotensin II and aldosterone regulation. Regul Pept (1999) 80:27–32 10.1016/S0167-0115(99)00004-X 10235631

[4] Unger T Blood pressure lowering and renin-angiotensin system blockade. J Hypertens Suppl (2003) 21:S3–7 10.1097/00004872-200307006-00002 14513945

[5] Peach MJ Renin-angiotensin system: biochemistry and mechanisms of action. Physiol Rev (1977) 57:313–70 191856 10.1152/physrev.1977.57.2.313

[6] Cushman DW Ondetti MA Inhibi tors of angiotensin-converting enzyme for treatment of hypertension. Biochem Pharmacol (1980) 29:1871–7 10.1016/0006-2952(80)90096-9 6249323

[7] Mahon JM Carr RD Nicol AK Henderson IW Angiotensin (1-7) is an antagonist at the type 1 angiotensin II receptor. J Hypertens (1994) 12:1377–81 10.1097/00004872-199412000-00010 7706697

[8] Chappell MC Emerging evidence for a functional angiotensin-converting enzyme 2-angiotensin-(1-7)-MAS receptor axis: more than regulation of blood pressure? Hypertension (2007) 50:596–9 10.1161/HYPERTENSIONAHA.106.076216 17785634

[9] Schindler C Bramlage P Kirch W Ferrario CM Role of the vasodilator peptide angiotensin-(1-7) in cardiovascular drug therapy. Vasc Health Risk Manag (2007) 3:125–37 17583183 PMC1994039

[10] Santos RA Ferreira AJ Verano-Braga T Bader M Angiotensin-converting enzyme 2, angiotensin-(1-7) and Mas: new players of the renin-angiotensin system. J Endocrinol (2013) 216:R1–17 10.1530/JOE-12-0341 23092879

[11] Capettini LS Montecucco F Mach F Stergiopulos N Santos RA DA Silva RF Role of renin-angiotensin system in inflammation, immunity and aging. Curr Pharm Des (2012) 18:963–70 10.2174/138161212799436593 22283774

[12] Li YH Jiao LH Liu RH Chen XL Wang H Wang WH Localization of angiotensin II in pig ovary and its effects on oocyte maturation in vitro. Theriogenology (2004) 61:447–59 10.1016/S0093-691X(03)00246-2 14662143

[13] Danilczyk U Eriksson U Oudit GY Penninger JM Physiological roles of angiotensin-converting enzyme 2. Cell Mol Life Sci (2004) 61: 2714–9 10.1007/s00018-004-4241-6 15549172 PMC11924509

[14] Danilczyk U Penninger JM Angiotensin-converting enzyme II in the heart and the kidney. Circ Res (2006) 98:463–71 10.1161/01.RES.0000205761.22353.5f 16514079

[15] Vinson GP Teja R Ho MM Hinson JP Puddefoot JR The role of the tissue renin-angiotensin system in the response of the rat adrenal to exogenous angiotensin II. J Endocrinol (1998) 158:153–9 10.1677/joe.0.1580153 9771458

[16] Vinson GP Teja R Ho MM Hinson JP Puddefoot JR Role of the tissue renin-angiotensin system in the response of the rat adrenal to exogenous angiotensin II. Endocr Res (1996) 22:589–93 8969916 10.1080/07435809609043751

[17] Brunswig-Spickenheier B Mukhopadhyay AK Local regulatory factors in regulation of ovarian function: role of prorenin-renin-angiotensin-system. Indian J Exp Biol (2003) 41:669–81 15255370

[18] Yoshimura Y The ovarian renin-angiotensin system in reproductive physiology. Front Neuroendocrinol (1997) 18:247–91 10.1006/frne.1997.0152 9237079

[19] Gallagher PE Tallant EA Inhibition of human lung cancer cell growth by angiotensin-(1-7). Carcinogenesis (2004) 25:2045–52 10.1093/carcin/bgh236 15284177

[20] Jaiswal N Diz DI Tallant EA Khosla MC Ferrario CM Characterization of angiotensin receptors mediating prostaglandin synthesis in C6 glioma cells. Am J Physiol (1991) 260:R1000–6 2035693 10.1152/ajpregu.1991.260.5.R1000

[21] Kobayashi S Moriya H Nakabayashi I Nishiyama J Fukuda T Angiotensin II and IGF-I may interact to regulate tubulointerstitial cell kinetics and phenotypic changes in hypertensive rats. Hypertens Res (2002) 25:257–69 10.1291/hypres.25.257 12047042

[22] Pereira VM Reis FM Santos RA Cassali GD Santos SH Honorato-Sampaio K Gonadotropin stimulation increases the expression of angiotensin-(1-7) and MAS receptor in the rat ovary. Reprod Sci (2009) 16:1165–74 10.1177/1933719109343309 19703990 PMC7101720

[23] Pountain SJ Pipkin FB Hunter MG The ontogeny of components of the renin-angiotensin system in the porcine fetal ovary. Anim Reprod Sci (2010) 117:119–26 10.1016/j.anireprosci.2009.03.006 19372013

[24] Yoshimura T Ito M Matsui K Okamura H Effects of highly purified eicosapentaenoic acid on vascular reactivity to angiotensin II and norepinephrine in pregnant rabbits. Artery (1997) 22:242–50 9209697

[25] Ferreira R Gasperin B Santos J Rovani M Santos RA Gutierrez K Angiotensin II profile and mRNA encoding RAS proteins during bovine follicular wave. J Renin Angiotensin Aldosterone Syst (2011) 12:475–82 10.1177/1470320311403786 21459786

[26] Giometti IC Bertagnolli AC Ornes RC da Costa LF Carambula SF Reis AM Angiotensin II reverses the inhibitory action produced by theca cells on bovine oocyte nuclear maturation. Theriogenology (2005) 63:1014–25 10.1016/j.theriogenology.2004.05.022 15710189

[27] Erman A Chen-Gal B van Dijk DJ Sulkes J Kaplan B Boner G Ovarian angiotensin-converting enzyme activity in humans: relationship to estradiol, age, and uterine pathology. J Clin Endocrinol Metab (1996) 81:1104–7 10.1210/jc.81.3.1104 8772583

[28] Shuttleworth G Hunter MG Robinson G Broughton Pipkin F Immunocytochemical localization of angiotensin II receptor subtypes 1 and 2 in the porcine fetal, prepubertal and postpubertal ovary. J Anat (2002) 201:267–74 10.1046/j.1469-7580.2002.00091.x 12363277 PMC1570909

[29] Obermuller N Gentili M Gauer S Gretz N Weigel M Geiger H Immunohistochemical and mRNA localization of the angiotensin II receptor subtype 2 (AT2) in follicular granulosa cells of the rat ovary. J Histochem Cytochem (2004) 52:545–8 10.1177/002215540405200413 15034006

[30] de Gooyer TE Skinner SL Wlodek ME Kelly DJ Wilkinson-Berka JL Angiotensin II influences ovarian follicle development in the transgenic (mRen-2)27 and Sprague-Dawley rat. J Endocrinol (2004) 180:311–24 10.1677/joe.0.1800311 14765984

[31] Tanaka M Ohnishi J Ozawa Y Sugimoto M Usuki S Naruse M Characterization of angiotensin II receptor type 2 during differentiation and apoptosis of rat ovarian cultured granulosa cells. Biochem Biophys Res Commun (1995) 207:593–8 10.1006/bbrc.1995.1229 7864848

[32] Kotani E Sugimoto M Kamata H Fujii N Saitoh M Usuki S Biological roles of angiotensin II via its type 2 receptor during rat follicle atresia. Am J Physiol (1999) 276:E25–33 9886947 10.1152/ajpendo.1999.276.1.E25

[33] Miyamoto A Shirasuna K Sasahara K Local regulation of corpus luteum development and regression in the cow: impact of angiogenic and vasoactive factors. Domest Anim Endocrinol (2009) 37: 159–69 10.1016/j.domaniend.2009.04.005 19592192

[34] de la Chica-Rodriguez S Cortes-Denia P Ramirez-Exposito MJ De Saavedra JM Sanchez-Agesta R Perez Mdel C Doxazosin blockade of alpha 1-adrenergic receptors increases rat serum progesterone levels: a putative role of ovarian angiotensin III in steroidogenesis. Fertil Steril (2007) 88:1071–5 10.1016/j.fertnstert.2006.12.021 17445810

[35] de la Chica-Rodriguez S Cortes-Denia P Ramirez-Exposito MJ Martinez-Martos JM Effects of alpha1-adrenergic receptor blockade by doxazosin on renin-angiotensin system-regulating aminopeptidase and vasopressin-degrading activities in male and female rat thalamus. Horm Metab Res (2007) 39:813–7 10.1055/s-2007-991168 17992636

[36] Kasum M New insights in mechanisms for development of ovarian hyperstimulation syndrome. Coll Antropol (2010) 34: 1139–43 20977119

[37] Herr D Fraser HM Konrad R Holzheu I Kreienberg R Wulff C Human chorionic gonadotropin controls luteal vascular permeability via vascular endothelial growth factor by down-regulation of a cascade of adhesion proteins. Fertil Steril (2013) 99:1749–58 10.1016/j.fertnstert.2013.01.120 23465821

[38] Reis FM Bouissou DR Pereira VM Camargos AF Dos Reis AM Santos RA Angiotensin-(1-7), its receptor Mas, and the angiotensin-converting enzyme type 2 are expressed in the human ovary. Fertil Steril (2011) 95:176–81 10.1016/j.fertnstert.2010.06.060 20674894

[39] Costa AP Fagundes-Moura CR Pereira VM Silva LF Vieira MA Santos RA Angiotensin-(1-7): a novel peptide in the ovary. Endocrinology (2003) 144:1942–8 10.1210/en.2002-220787 12697701

[40] Goncalves PB Ferreira R Gasperin B Oliveira JF Role of angiotensin in ovarian follicular development and ovulation in mammals: a review of recent advances. Reproduction (2012) 143:11–20 10.1530/REP-11-0192 22046052

[41] Hayashi K Miyamoto A Berisha B Kosmann MR Okuda K Schams D Regulation of angiotensin II production and angiotensin receptors in microvascular endothelial cells from bovine corpus luteum. Biol Reprod (2000) 62:162–7 10.1095/biolreprod62.1.162 10611081

[42] Davis JS Rueda BR Spanel-Borowski K Microvascular endothelial cells of the corpus luteum. Reprod Biol Endocrinol (2003) 1:89 10.1186/1477-7827-1-89 14613535 PMC305343

[43] Bokal EV Vrtovec HM Virant Klun I Verdenik I Prolonged HCG action affects angiogenic substances and improves follicular maturation, oocyte quality and fertilization competence in patients with polycystic ovarian syndrome. Hum Reprod (2005) 20:1562–8 10.1093/humrep/deh789 15734758

[44] Rizzo A Minoia G Trisolini C Mutinati M Spedicato M Manca R Renin and ovarian vascularization in cows with follicular cysts after epidural administration of a GnRH analogue. Anim Reprod Sci (2009) 116:226–32 10.1016/j.anireprosci.2009.02.016 19361938

[45] Schams D Berisha B Neuvians T Amselgruber W Kraetzl WD Real-time changes of the local vasoactive peptide systems (angiotensin, endothelin) in the bovine corpus luteum after induced luteal regression. Mol Reprod Dev (2003) 65:57–66 10.1002/mrd.10257 12658634

[46] Schauser KH Nielsen AH Winther H Dantzer V Poulsen K Localization of the renin-angiotensin system in the bovine ovary: cyclic variation of the angiotensin II receptor expression. Biol Reprod (2001) 65:1672–80 10.1095/biolreprod65.6.1672 11717127

[47] Herr D Duncan WC Hack G Konrad R Kreienberg R Wulff C Regulated expression of the renin-angiotensin-system in human granulosa lutein cells: angiotensin II increases VEGF expression but its synthesis is reduced by hCG. Arch Gynecol Obstet (2010) 281: 409–16 10.1007/s00404-009-1135-8 19521712

[48] Anton L Brosnihan KB Systemic and uteroplacental renin – angiotensin system in normal and pre-eclamptic pregnancies. Ther Adv Cardiovasc Dis (2008) 2:349–62 10.1177/1753944708094529 19124433 PMC2692864

[49] Cooper AC Robinson G Vinson GP Cheung WT Broughton Pipkin F The localization and expression of the renin-angiotensin system in the human placenta throughout pregnancy. Placenta (1999) 20:467–74 10.1053/plac.1999.0404 10419812

[50] Kalenga MK Thomas K DE Gasparo M DE Hertogh R Determination of renin, angiotensin converting enzyme and angiotensin II levels in human placenta, chorion and amnion from women with pregnancy induced hypertension. Clin Endocrinol (Oxf) (1996) 44:429–33 10.1046/j.1365-2265.1996.703525.x 8706309

[51] Li X Shams M Zhu J Khalig A Wilkes M Whittle M Cellular localization of AT1 receptor mRNA and protein in normal placenta and its reduced expression in intrauterine growth restriction. Angiotensin II stimulates the release of vasorelaxants. J Clin Invest (1998) 101:442–54 10.1172/JCI119881 9435317 PMC508584

[52] Shah DM Banu JM Chirgwin JM Tekmal RR Reproductive tissue renin gene expression in preeclampsia. Hypertens Pregnancy (2000) 19:341–51 10.1081/PRG-100101996 11118408

[53] Pan N Frome WL Dart RA Tewksbury D Luo J Expression of the renin-angiotensin system in a human placental cell line. Clin Med Res (2013) 11:1–6 10.3121/cmr.2012.1094 22997355 PMC3573093

[54] Ito M Itakura A Ohno Y Nomura M Senga T Nagasaka T Possible activation of the renin-angiotensin system in the feto-placental unit in preeclampsia. J Clin Endocrinol Metab (2002) 87:1871–8 10.1210/jc.87.4.1871 11932332

[55] Anton L Merrill DC Neves LA Stovall K Gallagher PE Diz DI Activation of local chorionic villi angiotensin II levels but not angiotensin (1-7) in preeclampsia. Hypertension (2008) 51:1066–72 10.1161/HYPERTENSIONAHA.107.103861 18259034 PMC2705753

[56] Herse F Dechend R Harsem NK Wallukat G Janke J Qadri F Dysregulation of the circulating and tissue-based renin-angiotensin system in preeclampsia. Hypertension (2007) 49:604–11 10.1161/01.HYP.0000257797.49289.71 17261642

[57] Yagami H Kurauchi O Murata Y Okamoto T Mizutani S Tomoda Y Expression of angiotensin-converting enzyme in human placenta and its physiologic role in the fetal circulation. Obstet Gynecol (1994) 84:453–7 8058247

[58] Neves LA Stovall K Joyner J Valdes G Gallagher PE Ferrario CM ACE2 and ANG-(1-7) in the rat uterus during early and late gestation. Am J Physiol Regul Integr Comp Physiol (2008) 294: R151–61 10.1152/ajpregu.00514.2007 17977916

[59] Li JS Touyz RM Schiffrin EL Effects of AT1 and AT2 angiotensin receptor antagonists in angiotensin II-infused rats. Hypertension (1998) 31:487–92 10.1161/01.HYP.31.1.487 9453350

[60] Knock GA Sullivan MH Mccarthy A Elder MG Polak JM Wharton J Angiotensin II (AT1) vascular binding sites in human placentae from normal-term, preeclamptic and growth retarded pregnancies. J Pharmacol Exp Ther (1994) 271:1007–15 7965763

[61] Petit A Geoffroy P Belisle S Expression of angiotensin II type-I receptor and phospholipase C-linked G alpha q/11 protein in the human placenta. J Soc Gynecol Investig (1996) 3: 316–21 10.1016/S1071-5576(96)00035-4 8923415

[62] Kalenga MK DE Gasparo M DE Hertogh R Whitebread S Vankrieken L Thomas K [Angiotensin II receptors in the human placenta are type AT1]. Reprod Nutr Dev (1991) 31:257–67 10.1051/rnd:19910307 1878151

[63] Pringle KG Tadros MA Callister RJ Lumbers ER The expression and localization of the human placental prorenin/renin-angiotensin system throughout pregnancy: roles in trophoblast invasion and angiogenesis? Placenta (2011) 32: 956–62 10.1016/j.placenta.2011.09.020 22018415

[64] Wang Y Pringle KG Chen YX Zakar T Lumbers ER Regulation of the renin-angiotensin system (RAS) in BeWo and HTR-8/SVneo trophoblast cell lines. Placenta (2012) 33:634–9 10.1016/j.placenta.2012.05.001 22647832

[65] Johnson MC Castro A Troncoso JL Vantman D Devoto L Vega M Presence of angiotensin II and expression of angiotensin II type-2 receptor in human fallopian tube. Fertil Steril (1998) 70:740–6 10.1016/S0015-0282(98)00254-4 9797108

[66] Pawlikowski M Melen-Mucha G Mucha S The involvement of the renin-angiotensin system in the regulation of cell proliferation in the rat endometrium. Cell Mol Life Sci (1999) 55:506–10 10.1007/s000180050307 10228564 PMC11146930

[67] Vaz-Silva J Carneiro MM Ferreira MC Pinheiro SV Silva DA Silva-Filho AL The vasoactive peptide angiotensin-(1-7), its receptor Mas and the angiotensin-converting enzyme type 2 are expressed in the human endometrium. Reprod Sci (2009) 16:247–56 10.1177/1933719108327593 19164480

[68] Li XF Ahmed A Dual role of angiotensin II in the human endometrium. Hum Reprod (1996) 11(Suppl 2):95–108 10.1093/humrep/11.suppl_2.95 8982751

[69] Matsumoto T Sagawa N Mukoyama M Tanaka I Itoh H Goto M Type 2 angiotensin II receptor is expressed in human myometrium and uterine leiomyoma and is down-regulated during pregnancy. J Clin Endocrinol Metab (1996) 81:4366–72 10.1210/jc.81.12.4366 8954043

[70] Shah DM Higuchi K Inagami T Osteen KG Effect of progesterone on renin secretion in endometrial stromal, chorionic trophoblast, and mesenchymal monolayer cultures. Am J Obstet Gynecol (1991) 164:1145–50 10.1016/0002-9378(91)90603-O 2014841

[71] Deshayes F Nahmias C Angiotensin receptors: a new role in cancer? Trends Endocrinol Metab (2005) 16:293–9 10.1016/j.tem.2005.07.009 16061390

[72] Sullivan MJ Hasser EM Moffitt JA Bruno SB Cunningham JT Rats exhibit aldosterone-dependent sodium appetite during 24 h hindlimb unloading. J Physiol (2004) 557:661–70 10.1113/jphysiol.2004.062265 15047775 PMC1665085

[73] Chappell S Morgan L Searching for genetic clues to the causes of pre-eclampsia. Clin Sci (Lond) (2006) 110:443–58 10.1042/CS20050323 16526948

[74] Laskowska M Vinson GP Szumilo J Laskowska K Leszczynska-Gorzelak B Oleszczuk J Comparative analysis of the angiotensin-II receptor in placental vascular endothelial cells in preeclamptic and normotensive patients. Gynecol Obstet Invest (2003) 56:55–60 10.1159/000072704 12897464

[75] Anton L Merrill DC Neves LA Diz DI Corthorn J Valdes G The uterine placental bed renin-angiotensin system in normal and preeclamptic pregnancy. Endocrinology (2009) 150: 4316–25 10.1210/en.2009-0076 19520788 PMC2736074

[76] Sykes SD Pringle KG Zhou A Dekker GA Roberts CT Lumbers ER Fetal sex and the circulating renin-angiotensin system during early gestation in women who later develop preeclampsia or gestational hypertension. J Hum Hypertens (2013). 10.1038/jhh.2013.51 23782994

[77] Valdes G Neves LA Anton L Corthorn J Chacon C Germain AM Distribution of angiotensin-(1-7) and ACE2 in human placentas of normal and pathological pregnancies. Placenta (2006) 27: 200–7 10.1016/j.placenta.2005.02.015 16338465

[78] Song C Xie S Wang J Lian J Diao B Tang Y Association of angiotensinogen gene polymorphisms and angiogenic factors with preeclampsia in Chinese women. Gynecol Obstet Invest (2013) 76:64–8 10.1159/000352070 23860016

[79] Ino K Shibata K Kajiyama H Yamamoto E Nagasaka T Nawa A Angiotensin II type 1 receptor expression in ovarian cancer and its correlation with tumour angiogenesis and patient survival. Br J Cancer (2006) 94:552–60 10.1038/sj.bjc.6602961 16434990 PMC2361172

[80] Suganuma T Ino K Shibata K Kajiyama H Nagasaka T Mizutani S Functional expression of the angiotensin II type 1 receptor in human ovarian carcinoma cells and its blockade therapy resulting in suppression of tumor invasion, angiogenesis, and peritoneal dissemination. Clin Cancer Res (2005) 11:2686–94 10.1158/1078-0432.CCR-04-1946 15814650

[81] Song L Zhang SL Bai KH Yang J Xiong HY Li X Serum agonistic autoantibodies against type-1 angiotensin II receptor titer in patients with epithelial ovarian cancer: a potential role in tumor cell migration and angiogenesis. J Ovarian Res (2013) 6:22 10.1186/1757-2215-6-22 23561060 PMC3626713

[82] Shibata K Kikkawa F Mizokami Y Kajiyama H Ino K Nomura S Possible involvement of adipocyte-derived leucine aminopeptidase via angiotensin II in endometrial carcinoma. Tumour Biol (2005) 26:9–16 10.1159/000084181 15741767

[83] Watanabe Y Shibata K Kikkawa F Kajiyama H Ino K Hattori A Adipocyte-derived leucine aminopeptidase suppresses angiogenesis in human endometrial carcinoma via renin-angiotensin system. Clin Cancer Res (2003) 9:6497–503 14695154

[84] Freitas-Silva M Pereira D Coelho C Bicho M Lopes C Medeiros R Angiotensin I-converting enzyme gene insertion/deletion polymorphism and endometrial human cancer in normotensive and hypertensive women. Cancer Genet Cytogenet (2004) 155:42–6 10.1016/j.cancergencyto.2004.03.020 15527901

[85] Piastowska-Ciesielska AW Pluciennik E Wojcik-Krowiranda K Bienkiewicz A Nowakowska M Pospiech K Correlation between VEGFR-2 receptor kinase domain-containing receptor (KDR) mRNA and angiotensin II receptor type 1 (AT1-R) mRNA in endometrial cancer. Cytokine (2013) 61:639–44 10.1016/j.cyto.2012.11.017 23273598

[86] Choi CH Park YA Choi JJ Song T Song SY Lee YY Angiotensin II type I receptor and miR-155 in endometrial cancers: synergistic antiproliferative effects of anti-miR-155 and losartan on endometrial cancer cells. Gynecol Oncol (2012) 126:124–31 10.1016/j.ygyno.2012.04.020 22525818

